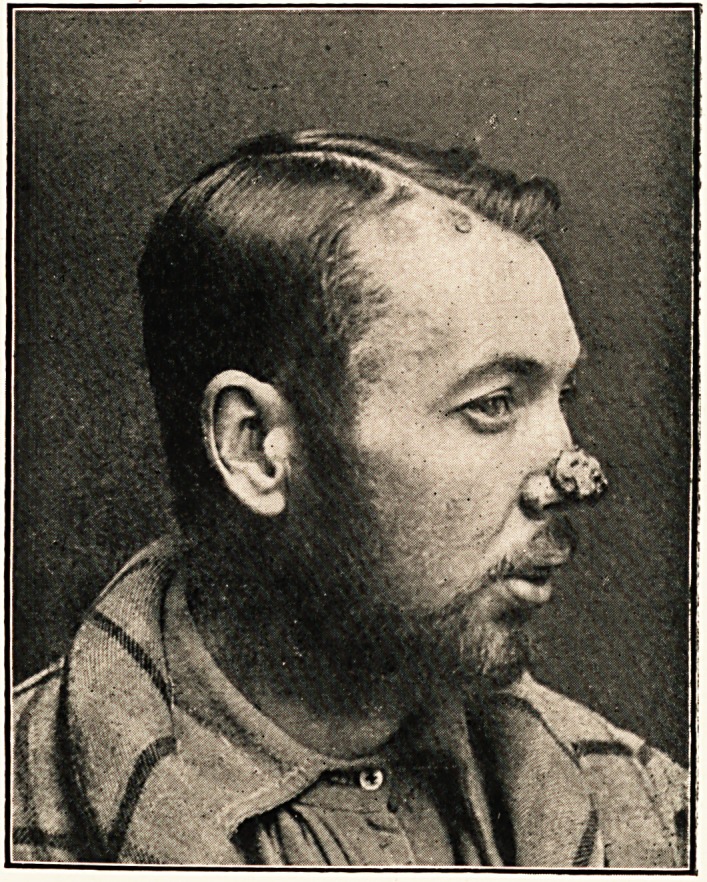# A Granuloma of the Nose, Due to Iodide of Potassium

**Published:** 1903-09

**Authors:** C. Percival Crouch

**Affiliations:** Honorary Surgeon to the Weston-Super-Mare Hospital.


### A GRANULOMA OF THE NOSE, DUE TO IODIDE OF POTASSIUM.

BY

C. Percival Crouch, M.B. Lond., F.R.C.S. Eng.,

*Honorary Surgeon to the Weston-super-Mare Hospital.*

---

C. B., aged 30, a groom, was admitted to the Weston-super-Mare Hospital on February 21st, 1902, suffering from specific disease of nose and larynx, and a granuloma on the tip of the nose.

*History of Present Illness.*—Thirteen months ago the patient was suddenly "seized with a violent cold in the head," with a profuse discharge of clear, somewhat offensive fluid from the nose. The discharge soon became fœtid and purulent, and pieces of bone were expelled from the nose from time to time: at the same time he suffered from violent pains in his head. Some six months ago two swellings appeared in the palate, which broke down, leaving perforations which persisted. For the last four months his voice has been growing hoarse, and lately has become a mere whisper; his hearing has also become very defective.

Antisyphilitic treatment was begun about a month ago. A week or ten days before admission some spots appeared on his forehead, like an iodide rash, and a small raised granuloma appeared on the tip of his nose, and rapidly increased in size, while similar growths presented themselves on forehead and temples. As the growth on nose grew rapidly worse, and the man appeared thoroughly ill and weak, he was admitted an in-patient.

*History of Previous Illness.*—Ten years ago, he says, he was treated for rheumatic fever, lasting six weeks; it was complicated with heart trouble, leaving permanent affection of aortic and mitral valves: at that time his hair came out very freely. Two years ago he was treated for gastric ulcer; six months ago, he says, he had "butterfly erysipelas." No history of syphilis could be elicited; but there is a slight scar on prepuce. He attributes his illness to his contact with a horse under his care, suffering from "greasy heels."

*Condition on Admission.*—The patient looked ill and cachectic when admitted. There was a very offensive purulent discharge from the nose, which showed, by the depression of the bridge, that much bone had been lost by disease. The granulomatous growth was entirely outside the nose, reaching from the edges of the nasal apertures upwards to the level of the nasal bones, and down the sides over the alæ nasi: it stopped abruptly at the junction of skin and mucous membrane; it was about one inch square, and raised about a quarter of an inch from the surface of the skin; it was covered by skin, and was quite firm in consistence—so firm, indeed, that it could not be scraped away with a Volkmann's spoon when considerable force was used; on pressure it exuded pus from numerous points, like a wet sponge; it was very vascular, and bled considerably when a small piece was cut away with a pair of scissors for diagnostic purposes. The temples exhibited somewhat similar growths, and there were smaller ones on the forehead: there were also some typical iodide spots on the face; there were no spots on any other part of the body. The voice was represented by a hoarse whisper, and breathing was noisy and laboured.

Laryngoscopic examination showed a large ulcerating swelling, like a breaking-down gumma, occupying the position of the left arytenoid and the left vocal cord, and very materially diminishing the space between the cords. The palate showed two perforations. The hearing was considerably impaired. The heart showed marked valvular disease, there being loud aortic and mitral murmurs present.

*Diagnosis.*—Although there was no ascertainable specific history, there was no doubt that the disease affecting the bones of the nose, the palate, and larynx was of syphilitic origin. The nature of the granuloma was not appreciated at first, and it was not until it was definitely ascertained that, while undoubted syphilitic lesions were improving under mercury and iodide, the granuloma was, if anything, growing bigger, that the iodide was discontinued and arsenic given instead, and caustic applied.

As I was at a loss to explain the nature of this growth, I sent some swabs up to the C. R. A., and a small piece of the growth to Dr. Andrewes and Dr. Drysdale, of St. Bartholomew's Hospital, who very kindly made some cultivations and inoculated a guinea-pig with some of the matter. They reported that there were no tubercle bacilli or glanders bacilli present: there were some bacilli corresponding to some diphtheritic organisms often found in the nose. The guinea-pig died in three to four days, after inoculation, of an acute spreading inflammation of the belly-wall, due to the presence of streptococci and pneumococci.

I was anxious to show the case to Mr. Hutchinson; but the man was so very ill, that I was not able to send him up to town. However, later on, when I showed him the photos and related the history of the case, he decided that the growth was due to potassium iodide on a syphilitic subject, and to a great extent increased by the existing heart lesion, which prevented the proper elimination of the iodide. Mr. Hutchinson also very kindly referred me to one of his own coloured pictures of a similar, but more extensive, affection, at the rooms of the

Polyclinic in Chenies Street. In this case the disease was much more pronounced and extensive: ulceration had taken place in many of the granulomata present in the face. The exhaustion consequent upon this condition has been so extreme as to reduce the patient to the lowest ebb of vitality. Some bromide rashes somewhat resemble this condition, and excellent coloured pictures of these may be seen at the Polyclinic. Mr. Hutchinson says that doubtless some cases of these iodide granulomata have been mistaken for granuloma fungoides, and it has also been mistaken for syphilis. In one case a patient had been taking iodide for a swelling in the groin, supposed to be syphilitic. The eruption had soon appeared, and had gone on steadily increasing, the specific remedy being pushed under the impression that the disease was syphilis.

*Treatment.*—The internal administration of arsenic was tried, but it had practically no effect upon the growth, and accordingly a saturated solution of chloride of zinc and collodion was applied to the growth: this caustic caused a big slough to separate, and a smooth, red, granulating surface was left, which slowly scarred over. The growth has not returned. His recovery was delayed by a severe attack of tachycardia; and as he had double aortic and mitral disease, it was feared he would not recover. However, he slowly became convalescent, and left the hospital. At the time of leaving, the nose presented a smooth, white, scarred surface at the seat of the former growth. The discharge from the nose was much less offensive. The laryngoscope showed that the gumma had healed, dragging down the epiglottis over the larynx, thereby much diminishing the entrance to the glottis.

*Remarks.*—The chief point to be noted about this decidedly rare condition is, that its nature is apt to be overlooked, and consequently the iodide to be continued. In this case the diagnosis was rendered more difficult by the fact that some undoubted iodide spots on the forehead disappeared, while the granuloma increased.

Acne due to iodide is apt to become tuberous. Iodide rashes are more apt to occur if there be either cardiac or renal disease present, causing a defect in elimination. An iodide rash may begin in twenty-four hours on pot. iod., gr. v. t. d. s.

The granuloma is a solid one, a copious cell-exudation pushing up the epidermis as a whole. In the College of Surgeons' Museum, in the Dermatological Section, there are some good representations of iodide granulomata :—

DRAWING NO. 30—*Exudations: Iodide Eruptions.*—A man with tuberous thickening of nose, face, and arm. (James Adams.)

Drawing No. 31—*Ditto.*—A man. On face, neck, and thorax are very prominent hemispherical inflammatory swellings, ulcerating; some as big as walnuts.

Drawing No. 28—*Ditto.*—A man, horsekeeper. Inflamed and elevated sores, which patient believed to be due to inoculation with "grease." (J. Hutchinson.)

This last case is of interest, inasmuch as this patient, too, attributed his condition to "grease," from which a horse under his care was suffering.

## Figures and Tables

**Figure f1:**